# Natural Antioxidants: Innovative Extraction and Application in Foods

**DOI:** 10.3390/foods10050937

**Published:** 2021-04-25

**Authors:** Monica Rosa Loizzo, Ana Sanches Silva

**Affiliations:** 1 Department of Pharmacy, Health and Nutritional Sciences, University of Calabria, 87036 Arcavacata di Rende, Italy; 2 National Institute for Agricultural and Veterinary Research (INIAV), I.P., Rua dos Lágidos, Lugar da Madalena, Vairão, 4485-655 Vila do Conde, Portugal; anateress@gmail.com or; 3 Center for Study in Animal Science (CECA), ICETA, University of Oporto, 4051-401 Oporto, Portugal

Research has devoted great attention to the study of the biological properties of plants, animal products, microorganisms, marine species, and fungi, among others, often driven by the need to discover new medicines. Many times, in order to enhance biological activities, extracts are prepared. One of the most well-studied biological activities is antioxidant capacity related to anticancer and antiaging properties, improvement of immune function, and protection against cardiovascular diseases and neurological disorders. Moreover, in foods, antioxidants allow for delayed oxidation onset and enhancing food shelf life.

Changes in lifestyle patterns and world population growth demand safe, nutritious, flavourful, colourful, affordable, and convenient food, and high-quality standards have increased the use of food additives, especially antioxidants. The effects of some food additives on human health are controversial, and synthetic food additives are often associated with potential public health risk. Therefore, there is a tendency to substitute synthetic food additives with natural compounds.

We have organized a Special Issue titled “Natural Antioxidants: Innovative Extraction and Application in Foods” in the Foods (ISSN 2304-8158; CODEN: FOODBV; https://www.mdpi.com/journal/foods, accessed on 24 April 2021). This thematic issue focused on the application of innovative extraction techniques for the recovery of natural antioxidants from foods and their possible application in food industries. This Special Issue, now converted into a book, includes 11 chapters, which are important contributions to this topic made by distinguished experts in this area. Ten of these chapters are research papers, and one is a review paper.

Chapter 1 is titled “The Effect of Blanching on Phytochemical Content and Bioactivity of *Hypochaeris* and *Hyoseris* Species (Asteraceae), Vegetables Traditionally Used in Southern Italy” [1]. This chapter regards the effect of blanching on the bioactivity and phytochemical content of *Hypochaeris* and *Hyoseris* species, traditionally used in Southern Italy. The results of this study indicated that these wild plants are a good source of bioactive compounds; however, their antioxidant capacity decreased after blanching. In fact, blanching water presented higher antioxidant capacity than the blanched samples. Therefore, the reuse of blanching water is recommended in food preparation because it is a good source of bioactives, and its consumption can increase the uptake of micronutrients.

Chapter 2 (“Effect of Microwave Pretreatment of Seeds on the Quality and Antioxidant Capacity of Pomegranate Seed Oil”) [2] is a very interesting study on the consequences of microwave pretreatment on pomegranate seeds and on the antioxidant capacity of pomegranate seed oil.

A considerable number of quality attributes were evaluated in three different pomegranate cultivars, including yellowness index, refractive index, oil yield, *p*-anisidine value, total oxidation value, conjugated dienes, total phenolic content, peroxide value, total carotenoid content, phytosterol composition, fatty acid composition, and antioxidant capacity through ferric reducing antioxidant power (FRAP) and 2,2-diphenyl-1-picrylhydrazyl (DPPH) radical scavenging capacity. Most of the parameters were increased after microwave pretreatment; however, punicic acid and beta-sitosterol were decreased.

Pomegranate seed oil may be enhanced if microwave pretreated seeds are used, although oil quality varies with cultivar.

Chapter 3 (“Chemical Composition and Antioxidant Activity of Thyme, Hemp, and Coriander Extracts: A Comparison Study of Maceration, Soxhlet, UAE, and RSLDE Techniques”) [3] is a valuable chapter comparing different extraction techniques for the obtainment of thyme, hemp, and coriander extracts. The selected techniques were maceration, Soxhlet, ultrasound-assisted extraction, and rapid solid–liquid dynamic extraction (RSLDE). Several parameters were measured in order to compare the extraction techniques. ABTS˙+, FRAP, and DPPH˙ assays were used to evaluate the antioxidant capacity. The total phenolic content by Folin–Ciocalteu method was also evaluated. The results revealed that all the evaluated techniques are valid extraction methods to extract bioactives and preserve their activity. However, extracts obtained by RSLDE showed to have slightly higher antioxidant capacity, and the technique is easy to use besides allowing the standardization of the extraction process.

Chapter 4 regards the paper “Organic Selenium as Antioxidant Additive in Mitigating Acrylamide in Coffee Beans Roasted via Conventional and Superheated Steam” [4]. In this chapter, the effect of coffee beans pretreated with selenium in the formation of acrylamide was evaluated in coffee beans roasted using two different methods (conventional versus superheated steam). The results showed that the antioxidant capacity of the organic selenium suppressed the formation of acrylamide during coffee roasting by 73%. Superheated steam roasting increased antioxidant activity and significantly reduced acrylamide (up to 32%), which was only noticed in the untreated coffee beans.

Chapter 5 is titled “Antioxidant Compounds for the Inhibition of Enzymatic Browning by Polyphenol Oxidases in the Fruiting Body Extract of the Edible Mushroom *Hericium erinaceus*” [5]. This noteworthy chapter focused on the identification of the cause of the dark brown pigmentation via the enzymatic reaction of the polyphenol oxidase (PPO) family with oxidation activity, and the reduction of the occurrence of this pigmentation. The mushroom contained relatively high amounts of natural antioxidant compounds for the inhibition of tyrosinase and the scavenging of free radicals. These antioxidants could diminish the browning reaction via PPO inhibitory mechanisms in the fruiting body of the *H. erinaceus* mushroom. 

These results of this chapter allow for understanding the metabolites and PPO enzymes responsible for the enzymatic browning reaction of *H. erinaceus*.

Chapter 6 comprises the paper “Impact of Stability of Enriched Oil with Phenolic Extract from Olive Mill Wastewaters” [6]. This notable chapter evaluated the effect of phenolic extract addition in the oxidative deterioration of sunflower oil. XAD-7-HP resin was used to recover phenolic compounds from olive mill wastewaters. The extract was evaluated in terms of single phenol concentration by ultra-high-performance liquid chromatography. The highest amount was found for hydroxytyrosol. The oxidation state of fortified sunflower oil was evaluated during 90 days for different physicochemical parameters (refractive index, peroxide value, and oxidative resistance to degradation) and antioxidant assays (DPPH, ABTS, and ORAC). The study revealed that there was an increase of 50% in the oxidative stability of fortified oil compared with control. This indicates that olive mill wastewaters can be valorized through an efficient extraction method. 

Chapter 7 concerns the paper “A Novel and Simpler Alkaline Hydrolysis Methodology for Extraction of Ferulic Acid from Brewer’s Spent Grain and Its (Partial) Purification through Adsorption in a Synthetic Resin” [7].

This chapter interestingly developed a simple method to extract ferulic acid (FA) from brewer’s spent grain (BSG), produced by brewing companies. The method includes an autoclave step to perform the alkaline hydrolysis, which allows for simplifying the postextraction process and increasing the ferulic acid yield. Finally, the extracted ferulic acid carries out a partial purification in a synthetic resin.

Chapter 8 is titled “Radical Scavenging and Antimicrobial Properties of Polyphenol Rich Waste Wood Extracts” [8]. This chapter evaluated the radical scavenging and antimicrobial capacities of wood waste extracts from black locust (*Robinia pseudoacacia* L.), mulberry (*Morus alba* L.), myrobalan plum (*Prunus cerasifera* Ehrh.), wild cherry (*Prunus avium* L.), and different species of oaks (*Quercus petraea* (Matt.) Liebl., *Q. robur* L., and *Q. cerris* L.) in order to conclude about their potential use in the food and pharmaceutical industries. Phenolic compounds were separated by using high-performance thin-layer chromatography (HPTLC), while radical scavenging activity was determined using DPPH-HPTLC. DPPH-HPTLC identified gallic, ferulic, and/or caffeic acids as the compounds with the highest contribution to antioxidant capacity. Regarding antimicrobial capacity, mulberry extract showed the lowest minimum inhibitory concentration (MIC) and minimum bactericidal concentration (MBC) values against methicillin-resistant *Staphylococcus aureus*. The growth rate of *Listeria monocytogenes* was significantly inhibited by extracts of myrobalan plum, wild cherry, and mulberry. *Candida albicans* showed poor sensitivity to the action of all extracts, with the exception of the wild cherry extract. *Escherichia coli* was less sensitive to the tested extracts.

The study concluded that due to their antimicrobial activities, cherry and mulberry wood extracts can be useful in preserving short-shelf-life foods.

Chapter 9 comprises the paper “Extract from Broccoli Byproducts to Increase Fresh Filled Pasta Shelf Life” [9]. This is a very interesting chapter that aims to find an alternative to chemical/conventional preservation strategies for fresh filled pasta. The idea was to evaluate the suitability of an extract from broccoli by-products for this purpose. The study monitored microbiological and sensory qualities besides phenolic compound content before and after in vitro digestion of pasta samples.

Results revealed that the shelf life of the natural extract increased by 18 days in comparison with control. The addition of the by-products’ extract to pasta increased phenolic content after in vitro digestion. Consequently, it was concluded that broccoli by-products could be valorized for obtaining extracts able to enhance shelf life and improve the nutritional content of fresh filled pasta.

Chapter 10 is titled “Bioactive Compounds from Norway Spruce Bark: Comparison among Sustainable Extraction Techniques for Potential Food Applications” [10]. This is a great chapter comparing different techniques to extract antioxidants from Norway spruce bark (*Picea abies* (L.) Karst), a wood industry waste. Supercritical fluid extraction (SFE), pressurized liquid extraction (PLE), and ultrasound-assisted extraction (UAE) were compared, and results showed that PLE, using ethanol as solvent, was the most effective method for extracting total flavonoid compounds, with the highest antioxidant capacity according to ABTS˙+. On the other hand, UAE extract contained the maximum phenolic concentration and the highest antioxidant capacity by FRAP. UAE revealed the greatest efficiency in the extraction of *trans*-resveratrol with ethanol 70% (*v*/*v*), therefore suggesting its potential to be used to record antioxidants to be further applied in the food and pharmaceutical industries.

Chapter 11 comprises the review titled “A New Insight on Cardoon: Exploring New Uses besides Cheese Making with a View to Zero Waste” [11]. This chapter is the only review of this Special Issue/book. It regards cardoon (*Cynara cardunculus* L.), which is a plant native to the Mediterranean area whose flowers are used in cheese making, as vegetal rennet. The aim of the review was to address the properties of cardoon leaves, considered a by-product of this crop, and discuss their potential uses. The findings indicated that cardoon leaves are recognized for their potential health benefits, (e.g., diuretic, hepatoprotective, choleretic, hypocholesterolemic, anticarcinogenic, and antibacterial properties), and they can have new potential uses. In particular, they can be used for the preparation of extracts to be incorporated into active food packaging. In sum, the chapter concluded that the new uses of cardoon leaves will contribute to zero waste of this crop.

The chapters of this book address the unequivocal importance of natural antioxidants. There is a plethora of matrices that can be used to obtain natural antioxidants, including different parts of plants (e.g., leaves, bark, seeds), food by-products, and fungi. In conclusion, the choice of extraction technique is critical in order to improve the biological properties, especially the antioxidant and antimicrobial capacities, of the extracts and is strictly related to their potential application in the food industry. Nowadays, the food industry is looking for environmentally friendly extraction procedures as well as extraction procedures that allow for upscaling from lab to industry. Finally, the choice of pretreatment and processing methods can also have a great influence on the antioxidant capacity of the extracts. 

## Abbreviations

ABTS˙+	2:20-azino-bis(3-ethylbenzothiazoline-6-sulphonic acid
DPPH˙	2,2-diphenyl-1-picrylhydrazyl
FRAP	ferric reducing antioxidant power
HPTLC	high-performance thin-layer chromatography
MBC	minimum bactericidal concentration
MIC	minimum inhibitory concentration
PLE	pressurized liquid extraction
PPO	polyphenol oxidase
SFE	supercritical fluid extraction
UAE	ultrasound-assisted extraction
